# Assessment of Vaccine Herd Protection: Lessons Learned From Cholera and Typhoid Vaccine Trials

**DOI:** 10.1093/infdis/jiab358

**Published:** 2021-07-17

**Authors:** Jacqueline Deen, John D Clemens

**Affiliations:** 1 Institute of Child Health and Human Development, National Institutes of Health, University of the Philippines, Manila, Philippines; 2 International Centre for Diarrhoeal Disease Research, Dhaka, Bangladesh; 3 Fielding School of Public Health, University of California Los Angeles, Los Angeles, California, USA

**Keywords:** cholera, oral cholera vaccine, typhoid fever, typhoid vaccine, vaccine herd protection

## Abstract

Vaccine herd protection is the extension of the defense conferred by immunization beyond the vaccinated to unvaccinated persons in a population, as well as the enhancement of the protection among the vaccinated, due to vaccination of the surrounding population. Vaccine herd protection has traditionally been inferred from observations of disease trends after inclusion of a vaccine in national immunization schedules. Rather than awaiting outcomes of widescale vaccine deployment, earlier-stage evaluation of vaccine herd protection during trials or mass vaccination projects could help inform policy decisions about potential vaccine introduction. We describe the components, influencing factors, and implications of vaccine herd protection and discuss various methods for assessing herd protection, using examples from cholera and typhoid vaccine studies.

Vaccines provide direct protection to vaccine recipients by activating an immune response against targeted infections. This protection occurs regardless of the level of vaccination of the surrounding population. When vaccines are rolled out in a community, there may be extension of vaccine protection beyond the vaccine recipients to unvaccinated persons, as well as enhancement of the protection among the vaccinated [1]. These vaccine herd effects are an essential component of many immunization programs [2, 3].

Vaccine population effects may result from vaccine herd immunity or vaccine herd protection, which we consider as separate entities, although these 2 terms are often used interchangeably [1, 4]. We define vaccine herd immunity as the protection of nonvaccinated persons resulting from their exposure and immune response to live vaccine organisms shed by vaccinees in their community, as with the oral polio vaccine [5]. Vaccine herd immunity, described as such, would apply only to live vaccines that induce shedding and does not depend on whether the target infection is transmitted from person to person, or another route. During the development of a candidate vaccine, regulatory authorities recommend that shedding data should be collected. There have been relatively few documented cases of vaccine-strain organisms infecting and eliciting immune responses in contacts of a vaccinated person, other than the oral polio vaccine.

Compared with vaccine herd immunity, vaccine herd protection is the more common mechanism of vaccine-induced population effects. Vaccine herd protection results from a decline in the transmission of a pathogen within a community when a sufficient proportion of the population has been immunized [1]. Vaccine herd protection may be induced by live or nonlive vaccines but occurs only for infections that are transmitted from person to person, either directly or indirectly. Cocooning, a strategy of vaccinating those in close contact with immunocompromised persons or infants too young to receive or mount a response to a vaccine [6], is a form of vaccine herd protection focused on especially vulnerable persons.

Vaccine herd protection has traditionally been inferred from observations of disease trends after inclusion of a vaccine in national immunization schedules. For example, introduction of a universal toddler hepatitis A immunization program in Israel resulted in an interruption of transmission and a decline in disease incidence in older age groups [7], elimination of *Haemophilus influenzae* type b (Hib) disease in The Gambia occurred after the implementation of routine childhood Hib conjugate vaccination [8], and pneumococcal conjugate immunization of infants in the United States extended protection to children too young to be immunized [9] and to older individuals [10].

Instead of waiting for impact assessments following widescale vaccine deployment, earlier-stage evaluation of vaccine herd protection during trials or mass vaccinations could help inform policy decisions about potential vaccine introduction [1]. Considerable experience has accumulated in the assessment of herd protection in cholera and typhoid vaccine studies. Although measures against cholera and typhoid fever have traditionally focused on prompt case management and improved access to safe water, sanitation, and hygiene, in recent years cholera and typhoid vaccines have become important additional tools for prevention and control [11, 12]. Herd protection amplifies the impact of cholera and typhoid fever vaccinations and obviates the need to vaccinate the entire population to control transmission [13]. The devastating effects of uncontrolled cholera outbreaks [14] and for typhoid fever, the rapid global increase in antimicrobial drug resistance [15], emphasize the importance of herd protection against these diseases. In this article we describe the components, influencing factors, and implications of vaccine herd protection and discuss various methods for assessing herd protection, using examples from cholera and typhoid vaccine studies.

## COMPONENTS, INFLUENCING FACTORS, AND IMPLICATIONS OF VACCINE HERD PROTECTION

Vaccine herd protection is manifested as vaccine preventive impact in a population over and above that expected from direct protection and level of vaccine coverage per se. The components of vaccine herd protection include that conferred to the unvaccinated in the population through decreased exposure to the pathogen (indirect protection), the enhanced defense of the vaccinated due to their proximity to other vaccinated persons (total protection), and the protection of the entire population, irrespective of the vaccination status of its individual members, due to the combination of indirect and total effects (overall protection) [1]. Unlike vaccine recipients whose protection is mediated through an immune response to the vaccine (direct immunity), individuals who are immunologically naive to the disease of interest and shielded by indirect protection alone remain fully susceptible to the disease, should they be exposed [16].

The level of vaccine herd protection in the population may be influenced by several factors, including the direct protection against symptomatic and asymptomatic disease conferred to vaccinees, preexisting immunity of the population, vaccine coverage, and the extent of community mixing and mobility [17]. There are several implications of vaccine herd protection. Some vaccines may be cost-effective only when the impact of herd protection is considered, as has been noted for the inactivated oral cholera vaccines [18]. The demonstration of herd protection, particularly for vaccines that confer moderate degrees of individual direct protection, could determine whether the use of such vaccines in populations will be sufficient for disease control. Even if the vaccine-induced herd protection from such vaccines is not sufficient to achieve disease elimination from the community, the reduction of the risk of infection in the population by lesser degrees of herd protection may be a worthwhile public health goal. In addition, herd protection may shield those in whom immunization is not possible, such as young children too young to mount an immune response to vaccines [19] and the immunocompromised [20]. When infection prevalence has been substantially reduced, vaccine herd protection may prevent the emergence and spread of variants of some pathogens [21].

On the other hand, vaccine herd protection can change disease epidemiology with potentially deleterious consequences, such as shift the average age of infection to adulthood. This shift may be of significance if severe clinical outcomes are greater when infection occurs at an older age. For example, when the transmission of rubella virus in the population declines as a result of vaccine herd protection to a degree that results in women of reproductive age remaining susceptible to the virus, this may lead to cases of congenital rubella syndrome [22]. Additionally, vaccine herd protection may exert selection pressure that results in serotype replacements. This is an on-going issue under observation in pneumococcal immunization programs [23]. These issues need to be taken into consideration when deciding on widescale vaccine deployment. There is also the so-called “free-rider” paradox wherein persons living in a community with high vaccine coverage, who themselves refuse to be vaccinated due to vaccine hesitancy or antivaccination sentiments, may ironically benefit from herd protection [4].

## ASSESSMENT OF VACCINE HERD PROTECTION USING EXAMPLES FROM CHOLERA AND TYPHOID VACCINE STUDIES

Vaccine herd protection may be assessed in cluster-randomized trials, individually randomized trials, and nonrandomized (observational) studies. Randomized trials are ideal to avoid bias but nonrandomized studies, which may be the only designs acceptable for ethical, logistic, and financial reasons, are also valuable to show the real-world impact of vaccination. We describe various methodologies below, illustrated with assessments of herd protection in cholera and typhoid vaccine studies.

### Cluster-Randomized Controlled Trials

In a cluster-randomized trial groups of individuals are randomized to receive the study vaccine (intervention clusters) or the control agent (control clusters), usually in a blinded manner [24]. The potential units of randomization for such trials are diverse and include workplaces, clinics, hospitals, schools, households, and entire villages. Some members of the clusters may choose not to receive the study vaccine or the control agent. Indirect and total protection is assessed by comparing subsamples within the clusters.

**Figure 1.**
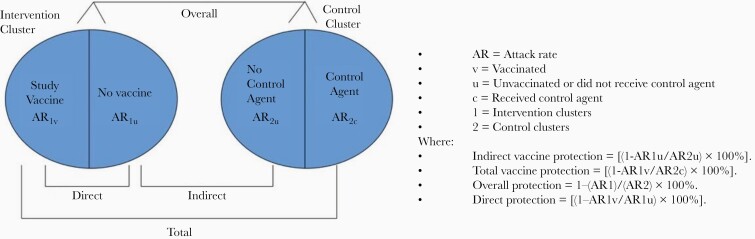
Evaluation of vaccine protection in cluster-randomization trials [17].

Indirect vaccine protection is estimated by comparing the rates of the disease of interest between nonvaccinated members of intervention clusters and nondosed members of the control clusters (Figure 1) [17]. Total vaccine protection is determined by comparing the rates among recipients of the study vaccine and recipients of the control agent. Overall vaccine protection is calculated by comparing rates among all members of the intervention versus all members of the control clusters. To avoid participation bias [25], those who receive the study vaccine are compared with those who receive the control agent (all study participants) to assess total protection. Similarly, those who chose not to receive the study vaccine are compared with those who chose not to receive the control agent (all nonparticipants) to assess indirect protection. In this way, the estimates are based on concurrent comparisons of groups that are similar by virtue of cluster randomization, which strengthens the credibility of inferences made from cluster-randomized trials [1].

A cluster-randomized trial was conducted in Kolkata, India, in which slum-dwellers who were 2 years of age or older were randomly assigned to receive a single dose of either typhoid Vi polysaccharide vaccine (intervention) or inactivated hepatitis A vaccine (control agent), according to geographic clusters, with 40 clusters in each of the 2 study arms [26]. The primary endpoint of the trial was the total Vi vaccine protection against typhoid fever when the vaccine was given under realistic public health conditions. The rate of typhoid episodes during 2 years of surveillance was compared across different groups to calculate the total, indirect, and overall vaccine protection (Table 1). During the 2 years of follow-up, total protection was 61% (95% confidence interval [CI], 41%–75%), indirect protection was 44% (95% CI, 2%–69%), and overall protection was 57% (95% CI, 37%–71%) [26, 27]. Vi coverage in the Vi clusters was only about 60%, making the population impact of overall protection equivalent to that for a vaccine with 100% direct protection but not conferring herd protection.

**Table 1.** Typhoid Vi Vaccine Effectiveness Estimates From a Cluster-Randomized Trial in Kolkata, India [26, 27]

Vaccine Protection	Number of Persons	Number of Typhoid Fever Episodes	Rate per 1000 Person-Years	% VE (95% CI)
Total				
Typhoid vaccine recipients	18 869	34	0.9	61 (41–75)
Hepatitis A vaccine recipients	18 804	96	2.7	*P* < .0001
Indirect				
Nonvaccinees in the intervention clusters	12 206	16	0.7	44 (2–69)
Nonvaccinees in the control clusters	12 877	31	1.3	*P* < .0429
Overall				
All residents in the intervention clusters	31 075	50	0.8	57 (37–71)
All residents in the control clusters	31 681	127	2.1	*P* < .0001

Abbreviations: CI, confidence interval; VE, vaccine effectiveness.

By contrast, a trial in Karachi, Pakistan with similar design and follow-up period but which vaccinated only children between the ages of 2 and 16 years found total protection of 57% (95% CI, 6%–81%) among children 5 to 16 years of age but none among children between the ages of 2 and younger than 5 years [28]. Furthermore, the study did not detect statistically significant indirect and overall vaccine protection. The difference in results between the sites has been ascribed to noninclusion of adults as vaccinees in the Karachi site, likely allowing continued transmission of typhoid fever in the intervention clusters.

The difference of outcome in these 2 cluster-randomized trials of Vi vaccine highlights the importance of study design [1, 25]. Ideally, the disease of interest should not be transmitted to a great extent by a group not targeted for vaccination within the clusters or into the clusters from adjacent populations. Such transmission could attenuate measured estimates of vaccine-induced herd effects. It seems likely, for example, that not vaccinating adults in the Karachi trial allowed considerable transmission of typhoid to continue in the clusters receiving typhoid vaccine. A second important consideration of cluster-randomized trials is that intercluster migration of participants should be minimal as this may change the vaccinee to nonvaccinee composition of the clusters and distort herd protection estimates. Third, careful consideration should be given to sample size; an adequate number of clusters are needed to prevent chance imbalances in baseline factors between vaccinated and control clusters and to allow for appropriate statistical inferences [29]. Typically, the sample size estimates for numbers of participants in cluster-randomized trials exceed those for individually randomized trials making the same assumptions about level of vaccine protection to be detected as well as types 1 and 2 errors.

#### Variations of Cluster-Randomized Trials to Assess Vaccine Herd Protection

##### Double-randomization design.

 Direct vaccine protection may be estimated using a cluster-randomized trial by comparing disease rates among the vaccinated and nonvaccinated members of the intervention clusters (Figure 1) [17]. But in conventional cluster-randomized trials, such comparisons may be subject to participation bias [30]. A hybrid design has been proposed to remedy this limitation, in which individuals in clusters randomly assigned to the vaccine are also individually randomly assigned to either the vaccine or the control agent, and direct protection is measured from the comparative attack rates in these vaccinees and controls [31]. However, this approach would likely add considerable complexity to the trial, and, to our knowledge, has not been used to date in vaccine field evaluations.

##### “Fried-egg” design.

 For valid estimates of vaccine herd protective effects, clusters should have little or no inward transmission of the disease of interest from the outside [32]. For many cluster-randomized trials this is not a major problem. However, in highly populated study sites (eg, urban slums) where isolation of clusters from inward transmission from the outside is not possible, the “fried-egg” approach may be useful. In this approach, the whole cluster receives the allocated vaccine or control agent but only the inner area of the cluster (the “egg yolk”) is included in the analysis or included in both the surveillance and analysis, while the “egg white” is the buffer zone [32]. For example, in the cluster-randomized typhoid Vi vaccine trial in Kolkata discussed above, during 2 years of follow-up, analysis of the entire clusters revealed that total protection was 61% (95% CI, 41%–75%), overall protection was 57% (95% CI, 37%–71%), and indirect protection was 44% (95% CI, 2%–69%) (Table 1) [26, 27]. In the innermost 25% of households of the clusters, total protection and overall protection were higher at 82% (95% CI, 48%–94%) and 66% (95% CI, 27%–84%), respectively; there was not a sufficient sample size to demonstrate such a trend for indirect protection [27]. The fried-egg approach helps ensure that the vaccine protection estimates in the inner area is less affected by spill-over, although the fact that the egg-white buffers for vaccinated clusters contain vaccinees, but those for control clusters do not, may theoretically lead to some exaggeration of the magnitude of estimates of vaccine herd protection.

### Individually Randomized Controlled Trials

Until recently, individually randomized trials were utilized only for measuring direct vaccine protection of vaccine recipients. However, if the individually randomized trial includes a sufficiently large range of vaccine coverage for different areas of the trial site, vaccine herd effects can be estimated by the correlation between incidence rates of the target disease in members of the geographic clusters, usually defined by geographic information systems (GIS), and the level of vaccine coverage in members of the surrounding cluster. In this design, illustrated by a reanalysis of a placebo-controlled, individually randomized trial of the inactivated oral cholera vaccine in Matlab, Bangladesh [33], advantage is taken of the differing levels of vaccine coverage of geographically defined groups of individuals that may occur by chance in the randomization process or due to differing participation rates. The geographic unit of analysis was the *bari*, which is a patrilineal-linked cluster of households, of which 6423 were included in the analysis. Most transmission of cholera is thought to occur within rather than between *baris*. The incidence rates of cholera among placebo recipients were inversely related to levels of vaccine coverage in and around the *baris* (7 cases per 1000 in the lowest quintile of coverage vs 1.5 cases per 1000 in the highest quintile; *P* < .0001) demonstrating that the oral cholera vaccine induces indirect protection of nonvaccinees [33]. A dynamic, population-based model of cholera transmission in Bangladesh, using information from the same trial, showed that if about half the population were vaccinated, this would reduce the number of cholera cases among unvaccinated people by 89% and among the entire population by 93% [13].

### Nonrandomized (Observational) Studies

GIS mapping may also be used in the assessment of herd protection in mass vaccinations given through public health programs. In the absence of a comparator group receiving the control agent or placebo, comparisons are made between the vaccinated and the nonvaccinated individuals in the community. The geographic area where the vaccine is deployed is virtually divided into clusters with a mixture of vaccinated and the nonvaccinated individuals. Indirect protection is estimated by comparing the disease incidence in nonvaccinated individuals in each geographic segment, by level of vaccine coverage [34]. Defining an appropriate geographic size of the virtual segments may need exploration of different sizes, unless discrete geographic units are already present.

For example, following a mass oral cholera vaccination campaign in Zanzibar, the incidence of acute watery diarrhea (laboratory confirmed as cholera or noncholera) over 14 months was assessed in vaccine recipients and nonrecipients [34]. Indirect protection was indicated by the subsequent lower risk of cholera in nonvaccinated individuals residing in areas with high vaccine coverage than in those residing in areas with low vaccine coverage. There were 2.29 cholera cases per 1000 in the lowest quintile of coverage versus 0.87 cases per 1000 in the highest quintile; *P* < .0001. In nonrandomized studies, a concurrently conducted bias-indicator or sham study may be incorporated to assess whether the results could be attributed to bias [35]. In a bias-indicator study, vaccine protection is assessed against another disease (detected using identical methods to identify the disease of interest) against which protection is not expected. In the Zanzibar cohort study, the absence of vaccine protection against non-cholera diarrhea suggested that the vaccine effectiveness found against cholera could not be explained by bias [34].

Oral cholera vaccine herd protection was also inferred from a study during the cholera epidemic in South Sudan in 2014 [36]. The daily cholera reproductive number among internally displaced persons living in settlements that had received oral cholera vaccination was <1 for most of the epidemic, compared to >1 in unvaccinated areas even though conditions were less suitable for transmission in these unvaccinated areas.

## DISCUSSION

Vaccine herd protection may be critical to the ability of a vaccine to control a disease under realistic public health conditions. Traditionally, herd protection by vaccines has been assessed through observations of disease trends after a vaccine is included in national immunization programs. For moderately protective vaccines, such as typhoid Vi vaccine and the inactivated oral cholera vaccines, consideration of herd protection may prove important to decisions about vaccine introduction, making assessment of herd protection critical. Vaccine herd protection is increasingly included in cost-effectiveness estimates for new vaccines. With the growing focus on the full public health value of vaccines, the assessment of herd protection may take on even greater importance in helping to determine stakeholder recommendations for vaccine uptake.

Innovative design and analytic methods allow the assessment of vaccine herd protection in both randomized and nonrandomized studies. We illustrate these methods from assessments of herd protection following oral cholera and typhoid vaccinations. The cluster-randomized trial design is the most straightforward method to assess vaccine herd protection and provides the most valid estimates, but may be challenging to implement and requires more complex approaches to sample size estimation and analysis. As well, it may not be possible to identify appropriate clusters for randomization. Incorporation of mapping techniques in individually randomized and nonrandomized studies offer alternative methods to assess vaccine herd protection. Exploiting newer approaches may offer improved information at an earlier stage to inform decisions on vaccine introduction.

## Notes

*Potential conflict of interests.* All authors: No reported conflicts of interest. All authors have submitted the ICMJE Form for Disclosure of Potential Conflicts of Interest. Conflicts that the editors consider relevant to the content of the manuscript have been disclosed.

Presented in part: 15th International Conference on Diarrheal Disease and Nutrition, Dhaka, Bangladesh, 28–30 January 2020.
